# Integrated Multi-Omics Analysis Uncovers Molecular Dysregulation in Glucosylceramide Synthase-Deficient Human Induced Pluripotent Stem Cells

**DOI:** 10.1016/j.mcpro.2026.101630

**Published:** 2026-07-23

**Authors:** Julia Beimdiek, Karsten Cirksena, Charlotte Rossdam, Smilla Brand-Merseburger, Astrid Oberbeck, Philippe V. Barbosa, Ana Tzvetkova, Malte Juchem, Christian Bär, Thomas Thum, Axel Schambach, Andreas Pich, Britta Brügger, Andreas W. Kuss, Falk F.R. Buettner

**Affiliations:** 1 Proteomics, Institute of Theoretical Medicine, University of Augsburg, Augsburg, Germany; 2 Institute of Clinical Biochemistry, Hannover Medical School, Hannover, Germany; 3 Cellular Proteome Research, Helmholtz Centre for Infection Research, Braunschweig, Germany; 4 Institute of Experimental Haematology, Hannover Medical School, Hannover, Germany; 5 Human Molecular Genetics Group, Department of Functional Genomics, Interfaculty Institute for Genetics and Functional Genomics, University Medicine Greifswald, Greifswald, Germany; 6 Institute of Bioinformatics, University Medicine Greifswald, Greifswald, Germany; 7 Institute of Molecular and Translational Therapeutic Strategies, Hannover Medical School, Hannover, Germany; 8 Institute of Toxicology, Hannover Medical School, Hannover, Germany; 9 Heidelberg University Biochemistry Center (BZH), Heidelberg, Germany

**Keywords:** Glycosphingolipids, Glucosylceramide-synthase UGCG, hiPSCs, Multi-omics, xCGE-LIF

## Abstract

Glycosphingolipids are membrane lipids characterized by highly diverse glycosylation patterns. They are involved in numerous cellular processes and can be linked to diseases. With the aim to investigate their role in early mammalian developmental processes *in vitro*, we knocked out the UDP-glucose ceramide glucosyltransferase gene (*UGCG*) encoding the enzyme glucosylceramide synthase (UGCG) in human induced pluripotent stem cells (hiPSCs) by CRISPR/Cas9. The functional impairment of UGCG was confirmed by glycomic profiling. UGCG KO hiPSCs displayed normal morphology, growth behavior, and expression of stem cell markers compared to WT hiPSCs. Furthermore, the KO cells maintained pluripotency, as evidenced by their capacity for *in vitro* differentiation into all three embryonic germ layers and their ability to form teratomas *in vivo*. Quantitative proteomic analysis of explanted teratoma derived from WT and UGCG KO hiPSCs revealed differential expression of numerous proteins, with notable enrichment of pathways associated with signal transduction or protein localization and transport. Phosphorylation profiling of key kinases and their targets uncovered reduced activation of STAT family transcription factors in UGCG KO hiPSCs. Complementary transcriptomic profiling combined with Ingenuity Pathway Analysis of differentially expressed genes between WT and UGCG KO hiPSCs, as well as their ectodermal derivatives, revealed a predicted positive enrichment of proteins associated with signaling pathways in UGCG KO hiPSCs. Global lipidomic profiling showed a significant increase in sphingomyelin levels in UGCG KO hiPSCs and their ectodermally differentiated derivatives, whereas total ceramide content remained comparable between UGCG KO and WT cells. In summary, despite the absence of overt phenotypic changes, our findings demonstrate that glycosphingolipid deficiency induces multiple molecular perturbations in hiPSCs.

## Highlights

• UGCG KO eliminates glucosylceramide-based glycosphingolipids.
• UGCG-deficient hiPSCs retain pluripotency and differentiation capacity.
• (Phospho)Proteomics reveals altered signaling and protein trafficking pathways.
• Sphingomyelin accumulates in UGCG-deficient cells.

## Graphical Abstract

**Figure:**
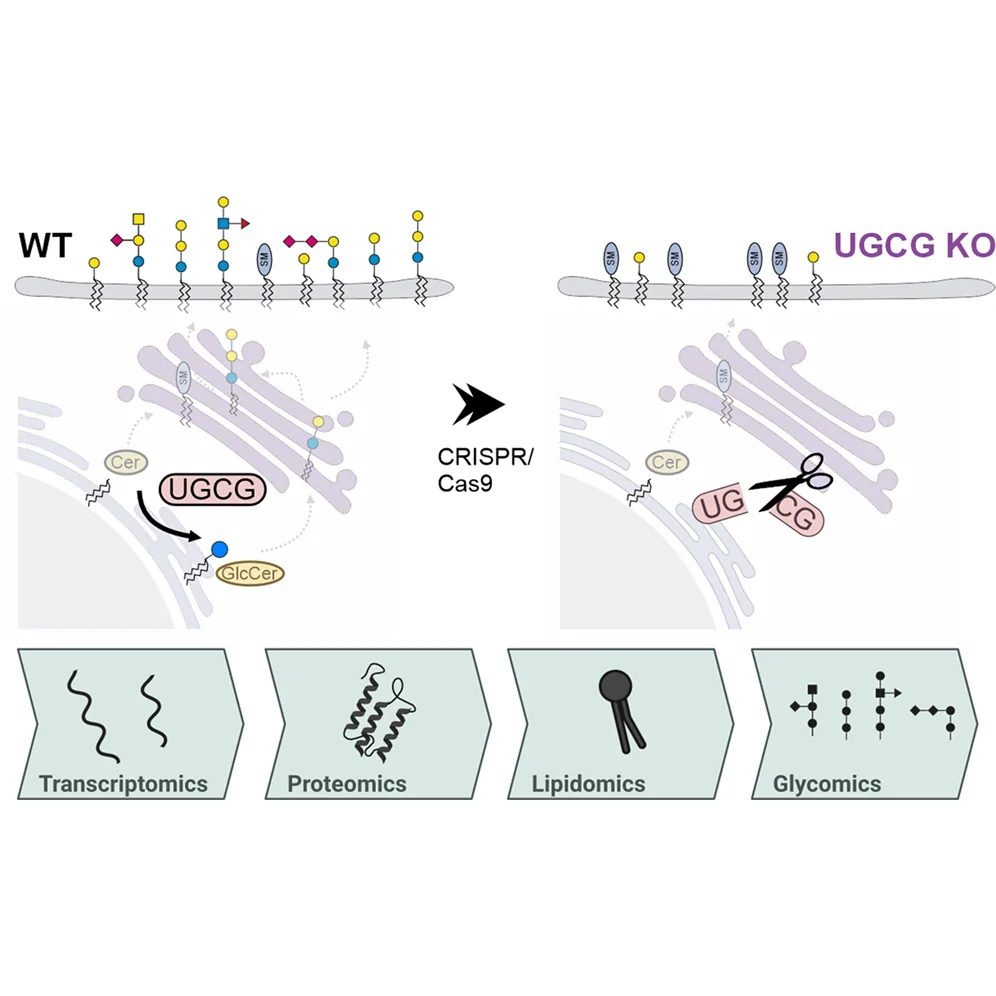

## In Brief

To define the role of glycosphingolipids in human pluripotent stem cells, we disrupted UGCG, the enzyme initiating glucosylceramide synthesis. Although KO cells retained self-renewal and developmental potential, integrated proteomic, phosphoproteomic, transcriptomic, and lipidomic analyses uncovered extensive molecular adaptations, including altered signaling networks, reduced STAT activity, and increased sphingomyelin levels. These findings reveal that glycosphingolipid depletion reshapes cellular homeostasis without producing overt phenotypic defects.

Glycosphingolipids (GSLs) are amphiphilic molecules ubiquitously present in mammalian cell membranes. They consist of a ceramide lipid moiety integrated into the membrane leaflet and a glycan head group predominantly exposed to the extracellular milieu (1). The glycan can be assembled from various monosaccharides, each chemically linked through distinct glycosidic bonds, leading to more than 400 different GSLs that are known to date; each with the potential for a specific biological function (2). Structural classification into globo-, ganglio-, and (neo) lacto-series is mainly based on the diversity of the glycan moiety; however, ceramide variations also add significant diversity. Embedded in the endomembrane system, GSLs are synthesized in a stepwise manner. After condensation of a sphingosine with acyl-CoA to ceramide, either galactose or glucose is transferred by activity of the galactosylceramide-synthase (UGT8) or UDP-glucose ceramide glucosyltransferase (UGCG), respectively (3). The majority of GSLs are built on glucosylceramide (GlcCer) and are widely distributed throughout the human body, whereas those derived from galactosylceramide (GalCer) exhibit a more restricted distribution pattern and are predominantly localized in the mammalian nervous system (4). GSLs are involved in diverse biological processes comprising cell–cell interactions, cell signaling, and intracellular trafficking processes (1, 2, 5, 6). They can assert their function on the same (*cis* regulation) or a neighboring cell (*trans* recognition) (1). GSLs play important roles in the formation of plasma membrane microdomains. These so-called lipid rafts present a distinct composition of lipids and proteins, differing from the rest of the plasma membrane, and are believed to be involved in various cellular processes (7). GSL synthesis is regulated in a cell type-specific manner and can be influenced by various factors, including nutrition and pathologies, making GSLs valuable biomarkers in health and disease.

Dysregulation of GSL metabolism can cause GSL-related diseases. These can be classified into glycosphingolipidoses which are genetically caused by specific enzyme deficiencies (*e*.*g*. Fabry, Gaucher, or Sandhoff disease) and peripheral neuropathies mainly caused by anti-ganglioside antibodies (like Guillain-Barrré, Miller-Fisher syndrome) (8). Several types of GSL-related diseases affect all organs; however, the central nervous system is primarily involved (9). Applying an UGCG-deficient mouse model, Yamashita *et al*. showed that mutant embryos proceeded into the gastrulation stage but developed abnormalities resulting in embryonic lethality at about E7.5 (10). Mutant embryos were smaller with reduced extraembryonic portion and the three major cavities were present but disorganized and reduced. Tunel assay revealed a large increase in apoptosis. Mice with conditional UGCG KO in neural cells exhibit structural and functional abnormalities in the cerebellum and peripheral nerves, leading to mortality within three weeks after birth (11). This underscores the essential role of GSLs in brain maturation. Throughout human brain development, for example, the quantity of sialylated GSLs (gangliosides) rises by approximately threefold from gestational weeks to the infant stage. Specifically, the levels of GM1 and GD1a are raised by up to 15-fold during this timeframe (12). GD3 has been demonstrated to be the primary GSL in embryonic brains and primary neural precursor cells (13, 14). However, KO mice deficient for the last enzymatic step of GD3 synthesis, exhibit no apparent phenotype except delayed nerve regeneration after intended lesioning (15). In addition, enterocyte-specific *Ugcg*-deficient mice demonstrated that GSLs play a primary role in intestinal endocytic function, but are not essential for brush border formation (16).

Given the limited knowledge of the molecular functions of GSLs in early human developmental processes, we established a CRISPR/Cas9-mediated UGCG KO model in human induced pluripotent stem cells (hiPSCs) that lacks GlcCer-based GSLs entirely. We performed comparative multi-omics analyses of WT and UGCG KO hiPSCs and derived neuroectodermal cells by glycomics, (phospho)proteomics, transcriptomics, and lipidomics. Our analyses revealed considerable changes at the molecular level.

## Experimental Procedures

### CRISPR/Cas9-Mediated Generation of UGCG KOs

With the aim to functionally inactivate the enzyme UGCG, we deleted a region of the coding sequence from the *UGCG* gene sequence in hiPSC Phoenix, applying the CRISPR/Cas9 system. The *UGCG* genomic sequence was screened using CCTop (17) to identify appropriate target sites for single guide RNAs (Supplemental Table S1). Cloning of single guide RNA/Cas9 vectors, plasmid electroporation, cell sorting based on GFP-expression, and single cell cultivation was performed as described previously (18). Resulting colonies were picked manually using a 200 μl pipette and transferred into Matrigel-coated 48-well plates. After cultivation for approximately one week, half of the cells were passaged into a new 48-well, while gDNA was extracted from the other half using proteinase K for clone genotyping using Phusion High-Fidelity PCR Kit (Thermo Fisher Scientific).

### Maintenance and Differentiation of hiPSCs

The hiPSC line HSC_Iso4_ADCF-iPC2 “Phoenix” (19) was cultured under standard conditions at 37 °C, 5% CO2, and 85% relative humidity. Phoenix hiPSCs were cultivated under feeder-free conditions as monolayer on hES-qualified Matrigel Matrix (Corning) and maintained in mTESR1 (STEMCEL Technologies,) or StemMACS iPS-Brew (Miltenyi Biotec) medium with daily medium changes. For passaging, cells were incubated in ReLeSR (STEMCELL Technologies), which was aspirated after 1 min and cells were further incubated for 7 min at 37 °C. For differentiations, cells were singularized using 0.5 mm EDTA in PBS for 12 min at 37 °C. After centrifugation at 300*g* for 3 min, cells were suspended in E8 medium (20) supplemented with 10 μm Rho-associated coiled-coil kinase (ROCK) inhibitor Y-27632 (STEMCELL Technologies) to enhance survival of single cells. A total of 1 × 10^6^ cells/well were seeded in Corning Costar Ultra-Low Attachment 6-well-plates (Merck). Endodermal differentiation was performed according to the protocol by Diekmann *et al*. (21). Briefly, seeding day was termed as d-1 and for initiation of differentiation (d0), medium was exchanged to RPMI 1640 medium (Biowest) supplemented with 1% (v/v) B27 minus insulin (Thermo Fisher Scientific), 5 μm CHIR99021 (STEMCELL Technologies), and 22 ng/ml Activin A (STEMCELL Technologies). Afterward, medium was exchanged to RPMI 1640 medium supplemented with 1% (v/v) B27 minus insulin and 22 ng/ml Activin A daily (d1-d3). Ectodermal differentiation was performed as previously described by Shi *et al*. (22). Medium was exchanged every day to neural induction medium supplemented with 10 μm SB431542 (Selleckchem) and 1 μm dorsomorphin (STEMCELL Technologies). Mesodermal differentiation in suspension culture was performed as described by Kempf *et al*. (23) with slight modifications. Briefly, seeding day was termed as d-4 and E8 medium was exchanged on d-2 and d-1 without ROCK inhibitor supplementation. For differentiation initiation (d0), medium was exchanged to RPMI 1640 medium supplemented with 2% (v/v) B27 minus insulin and 9 μm CHIR99021. After 27 h medium was exchanged to RPMI 1640 medium supplemented with 2% (v/v) B27 minus insulin and 5 μm IWP-4 (STEMCELL Technologies). On day 3, medium was replaced by RPMI 1640 medium supplemented with 2% (v/v) B27 minus insulin. On day 4 of differentiation, cells were singularized using 0.5 mm EDTA in PBS for 12 min at 37 °C.

### Flow Cytometry Analysis of Marker Protein Expression

Flow cytometric analyses targeting specific markers were performed to determine differentiation efficiencies on day 4 or to compare specific expression levels on iPSCs against day 7 (d7) ectodermal derivatives. For both approaches, singularized cells were washed with PBS and filtered using a 70 μm pre-separation filter (Miltenyi Biotec). Only for ectodermal differentiation efficiencies, cells were fixed with paraformaldehyde (4% (w/v) in PBS) for 20 min at 4 °C and subsequently permeabilized using 0.1% (v/v) Tween-20 in PBS for 15 min at 4 °C. Approximately 3 × 10^5^ cells were suspended in fluorescence-activated cell sorting (FACS) buffer (PBS with 1% (w/v) bovine serum albumin and 1 mm EDTA and probed with specific antibodies against CXCR-4 (endodermal), Nestin (ectodermal), NCAM and EpCAM (mesodermal), or respective isotype controls for 20 min at 4 °C. For direct comparison between iPSCs and d7 ectodermal derivatives, cells were stained against GalCer, sulfatide, TRA-1-60, SSEA-4, or respective isotype controls for 20 min at 4 °C. Detailed information about antibodies and dilutions can be found in Supplemental Table S4. After antibody labeling, cells were washed and resuspended in FACS buffer. Directly before measurement 1 ml PBS was added to the cell suspension. Analyses were performed using a CyFlow ML flow cytometer recording 10,000 cells per sample at a flow rate of 2 to 10 μl/s. Generated data were analyzed with FlowJo v10.7.2.

### Quantitative Polymerase Chain Reaction Analysis of Marker Gene Expression

RNA extraction with TRIzol (Invitrogen) and cDNA synthesis using RevertAid H Minus reverse transcriptase (Thermo Fisher Scientific) were performed according to the manufacturer’s instructions. Sequences of quantitative polymerase chain reaction primer (Sigma-Aldrich) are listed in Supplemental Table S3. Quantitative real-time PCR was performed on a QuantStudio 3 Real-Time PCR System (Thermo Fisher Scientific) using the SyGreen Lo-ROX Mix (Nippon Genetics Europe), employing dye intercalation for fluorescence detection. Generated raw data were analyzed by the Relative Quantification Module (Thermo Fisher Cloud) based on the ΔΔCT-method. Data sets were excluded from the analysis if the evaluation software assigned quality control flags indicating insufficient data quality.

### Glycan Profiling by xCGE-LIF Analysis

GSL-derived glycans were assessed as described previously by Rossdam *et al*. (24). Therefore, glycolipids were extracted from 2 × 10^6^ cells by chloroform/methanol extraction. LudgerZyme Ceramide Glycanase (Ludger) was used to release the glycan moiety of extracted glycolipids. *N*-glycans were analyzed as described previously by Beimdiek *et al*. (25) with slight modifications. After cell lysis and protein precipitation, a short SDS-PAGE allowed proteins to just enter the separation gel. The gel area containing the proteins was excised and *N*-glycans were released by in-gel digestion using peptide-*N*-glycosidase F (PNGaseF from *Elizabethkingia meningoseptica* 0.1 U/μl, Sigma-Aldrich). Subsequently, GSL-derived glycans and *N*-glycans were fluorescently labeled with 8-aminopyrene-1,3,6-trisulfonic acid trisodium salt (APTS, Sigma-Aldrich). A mixture of 2 μl APTS (20 mm in 3.5 M citric acid), 2 μl 2-picoline borane complex (2 M in dimethyl sulfoxide, Merck), and 2 μl water was incubated for exactly 16.5 h at 37 °C in darkness. After removal of excessive APTS by HILIC-SPE, labeled glycans were eluted in water and concentrated using a SpeedVac concentrator (Thermo Fisher Scientific). Samples were stored at −20 °C until further use. xCGE-LIF analyses were performed using an ABI PRISM 3130-Avant Genetic Analyzer (remodeled by advanced biolab service GmbH) operated with Run 3100-Avant Data Collection software 2.0. After injection of GSL-derived glycans for 15 s at 1.6 kV and a data delay of 50 s, data were collected for 30 min at 12 kV and 60 °C. For *N*-glycans, data were collected for 45 min at 15 kV and 60 °C. Resulting data were analyzed using the GeneMapperTM software v.3.7. Glycan annotation was performed using our in-house established glycan migration time databases.

### Teratoma Formation Assay

All animal experiments were approved by the Lower Saxony Office for Consumer Protection and Food Safety, under the application files 33.19-42502-04-19/3119 and 33.19-42502-04-15/2013 according to national and state law. hiPSCs were harvested with Accutase (PAN Biotech) and the cell number was adjusted to 2.5 × 10^6^ hiPSCs per injection. The harvested cells were centrifuged and resuspended in 75 μl hiPSC medium with 20 μm ROCK-inhibitor Y-27632 (Stemcell Technologies). To prepare the injection solution, 75 μl cooled Matrigel (Corning) was mixed into the cell suspension and the mixture was kept at 4 °C until injection. One teratoma per flank was induced in each NSG mouse by subcutaneous injection of hiPSCs. Mice were euthanized as soon as one teratoma reached a diameter of 1.5 cm. After isolation, teratoma were divided into two parts, with one part fixed overnight in 4% paraformaldehyde at 4 °C and used for H&E staining, and one part used for whole proteomic profiling.

### Proteomic Analysis of Teratoma by LC-MS

One half of each explanted teratoma was cryogenically grinded with a mortar and pestle upon freezing in liquid nitrogen. A total of 200 μl Laemmli buffer was added to one spade of pulverized sample and incubated for 5 min at 95 °C. A total of 150 μg of proteins of each teratoma were separated by SDS-PAGE and resulting gels were Coomassie stained. Gel lanes were divided into five fractions each, excised and individually digested with trypsin (Trypsin Gold, Mass Spectrometry Grade, Promega). The in-gel digestion protocol follows the procedure outlined by Shevchenko *et al*. (26). LC-MS analysis was performed by the Core Facility Proteomics (MHH) utilizing a Linear Trap Quadrupole Orbitrap-Velos mass spectrometer coupled to reverse-phase liquid chromatography system, following previously established methods (18).

### Processing of MS Data by MaxQuant and Perseus

The MaxQuant software suite v2.0.1.0 (27), utilizing the Andromeda search engine, processed raw data to identify and quantify proteins applying label-free quantification (LFQ). A comprehensive analysis of all 39 MS raw files was performed with default settings: specific digest mode, two missed cleavages, oxidation (M), and acetylation (N-term) as variable modifications, carbamidomethyl (C) as fixed modification, and mass tolerance set to 20 ppm (precursor) and 0.5 Da (MS/MS). MS spectra were matched against all Uniprot entries matching with “homo sapiens” (reviewed + unreviewed, 188,357 entries, downloaded on 07/01/2021) with a false discovery rate of 1% for proteins and peptides. Intensity-based absolute quantification and LFQ were utilized, with LFQ min. ratio count set to one and Fast LFQ disabled.

Subsequently, protein groups (output of MaxQuant analysis) were further analyzed using the Perseus software (28) version 1.6.15.0 with implemented annotations of the human proteome (mainPerseusAnnot.txt.gz downloaded on 07/22/2021 at http://annotations.perseus-framework.org). After exclusion of contaminants, proteins only identified by site, entries matching to the reverse database, and restriction to proteins identified in at least 75% of replicates in at least one biological group, LFQ values were normalized by transformation onto a logarithmic scale and missing values were replaced from normal distribution by imputation of low LFQ values (width: 0.3, down shift: 1.6). Protein levels of teratoma originating from different cell lines were compared by two-sample *t* test.

### Experimental Design and Statistical Rationale for Proteomic Analysis

For the MS-based proteomic analyses, four biological replicates per condition were analyzed. Therefore, four teratoma were generated from each hiPSC WT and UGCG KO1 cells. Cells were cultured under identical conditions and injected into eight independent mouse flanks. This design accounts for biological variability introduced during cell culture, *in vivo* teratoma formation, and the downstream analytical workflow. MS measurement was performed once, raw data were processed as described previously, and LFQ values were compared by two-sample *t* test with values <0.05 considered significant.

### Protein Phosphorylation Profiling Using a Phospho-Kinase Array Kit

To assess the phosphorylation state of a panel of human kinases, the Human Phospho-Kinase Array Kit (R&D systems) was used according to the manufacturer's protocol. Briefly, 1 × 10^7^ cells were harvested using 0.5 mm EDTA in PBS for 12 min at 37 °C, lysed in R&D lysis buffer at 4 °C for 30 min, and 1 mg protein was loaded per array set. Subsequently to the primary and horseradish peroxidase-conjugated secondary antibody incubation, the Chemi Reagent Mix was added directly before visualization *via* an Amersham Imager (8× binning and 10 s incrementals).

### Quantitative Transcriptomics by RNA Sequencing

For transcriptomic analysis of WT *versus* UGCG KO hiPSCs and their respective d7 ectodermal derivatives, four biological cell culture replicates were generated for each condition. From each sample, total RNA were isolated by TRIzol/chloroform extraction as described previously (29). Briefly, after RNA integrity testing, the NGS library was prepared using NEBNext Ultra II Directional RNA Library Prep Kit (NEB) in combination with NEBNext rRNA Deplection Kit (NEB). Sequencing was carried out on an Ilumina NextSeq platform (Ilumina) using NextSeq 500/550 High Output Kit v2.5 at 150 cycles (Ilumina). Resulting reads were mapped onto the human genome (GRCH38) using STAR v2.7.10b (30) with default parameters. For counting the reads matching genes, we utilized HTSeq-count script from the HTSeq suite v0.11.1 (31) with reverse strandedness to consider the used library kit. Applying DESeq2 (32), differentially expressed genes were identified.

Signaling pathway enrichment analysis was performed using QIAGEN Ingenuity Pathway Analysis (IPA) (QIAGEN Inc, https://digitalinsights.qiagen.com/IPA) (33). IPA is a bioinformatics platform that utilizes machine learning techniques to determine which signaling pathways are linked to the genes in a given dataset, drawing upon information contained within the Qiagen Knowledge Base. Fold changes and *p*-values for genes significantly regulated in both UGCG KO conditions *versus* WT were uploaded, and canonical pathways were analyzed. The resulting heatmaps display z-scores representing pathway activity predictions, with positive values indicating predicted activation and negative values indicating predicted inhibition.

### Quantitative Lipidomics by Mass Spectrometry

To analyze the lipid content of WT and *UGCG* KO hiPSCs, 3 × 10^7^ cells were harvested using EDTA. For the analysis of d7 derivatives, WT and *UGCG* KO hiPSCs were differentiated towards ectodermal lineage for 7 days. Cell pellets were resuspended in 1 ml ammonium bicarbonate:methanol (vol:vol, 1:1). Lipid extraction and MS analyses were performed as described previously (34). Cells were subjected to lipid extractions using an acidic liquid-liquid extraction method (35), except from plasmalogens, which were extracted under neutral conditions. In order to ensure that similar amounts of lipids were subjected to extractions, a test extraction was performed to determine the concentration of phosphatidylcholine (PC) as a bulk membrane lipid and to adapt extractions volumes to similar total lipid amounts. Typically, a total lipid amount of approximately 3000 pmol was subjected to extractions. Quantification was achieved by adding 1 to 3 internal lipid standards for each lipid class, with the standards resembling the structure of the endogenous lipid species. Lipid standards were added prior to extractions, using a master mix consisting of 75 pmol PC (14:1/14:1, 20:1/20:1, 22:1/22:1, Avanti Polar Lipids), 75 pmol sphingomyelin (SM, d18:1 with N-acylated 13:0, 17:0, 25:0, semi-synthesized (36)), 150 pmol deuterated cholesterol (D7-cholesterol, Avanti Polar Lipids), 42 pmol phosphatidylinositol (17:0/20:4, Avanti Polar Lipids), 37.5 pmol phosphatidylethanolamine and phosphatidylserine (both 14:1/14:1, 20:1/20:1, 22:1/22:1, semi-synthesized (36)), 37.5 pmol diacylglycerol (DAG, 15:0–18:1-D7, Avanti Polar Lipids), 37.5 pmol cholesteryl ester (9:0, 19:0, 24:1, Sigma-Aldrich), and 36 pmol triacylglycerol (TAG, D5-TG ISTD Mix I, Avanti Polar Lipids), 7.5 pmol Cer d18:1/18:0-D3, Matreya) and 7.5 pmol hexosylceramide (HexCer) (GlcCer d18:1/17:0, Avanti Polar Lipids), 7.5 pmol lactosylceramide (dihexosylceramide (Hex2Cer), d18:1/17:0, Avanti Polar Lipids), 15 pmol phosphatidic acid (17:0/20:4, Avanti Polar Lipids), 15 pmol phosphatidylglycerol (14:1/14:1, 20:1/20:1, 22:1/22:1, semi-synthesized (36)), and 7.5 pmol lysophosphatidylcholine (17:1, Avanti Polar Lipids). The phosphatidylethanolamine plasmalogen (PE P-) standard mix consisted of 33 pmol PE P-Mix 1 (16:0p/15:0, 16:0p/19:0, 16:0p/25:0), 46.5 pmol PE P- Mix 2 (18:0p/15:0, 18:0p/19:0, 18:0p/25:0), and 64.5 pmol PE P-Mix 3 (18:1p/15:0, 18:1p/19:0, 18:1p/25:0). Semi-synthesis of PE P- was performed as previously described (37). The final CHCl_3_ phase was evaporated under a gentle stream of nitrogen at 37 °C. Samples were either directly subjected to mass spectrometric analysis, or were stored at −20 °C prior to analysis, which was typically done within 1 to 2 days after extraction. Lipid extracts were resuspended in 10 mm ammonium acetate in 60 μl methanol. A total of 2 μl aliquots of the resuspended lipid extracts were diluted 1:10 in 10 mm ammonium acetate in methanol in 96-well plates (Eppendorf twin tec 96) prior to measurement. For cholesterol determinations, the remaining lipid extract was again evaporated and subjected to acetylation as previously described (38). Samples were analyzed on a QTRAP 6500+ mass spectrometer (Sciex) with chip-based (HD-D ESI Chip, Advion Biosciences) electrospray infusion and ionization *via* a Triversa Nanomate (Advion Biosciences) as described. Measurement of DAG and TAG was performed on a Q Exactive (Thermo Fisher Scientific). Here, 5 μl aliquots of the reconstituted lipid extracts were diluted with 15 μl 7.5 mm ammonium formate in isopropanol:methanol:chloroform (v:v:v, 4:2:1). MS settings and scan procedures are listed in Supplemental Table S4. Data evaluation was done using LipidView 1.3 beta (Sciex) for QTRAP 5500 data or LipidXplorer 1.2.8.1 (39, 40) and PeakStrainer for Q Exactive data (41), and in-house-developed software (ShinyLipids and ShinyLipidcountr_alpha4). The amount for endogenous molecular lipid species was calculated based on the intensities of the internal lipid standards.

## Results

### Genetic Deletion of UGCG Does Not Interfere with Common Hallmarks of Pluripotency in hiPSCs

To elucidate the relevance of GSLs in early mammalian developmental processes *in vitro*, we developed a GSL-deficient hiPSC model. Therefore, the CRISPR/Cas9 system was applied to mutate the *UGCG* in the hiPSC line Phoenix (19). To distinguish between UGCG-specific phenotypes and potential off-target effects of the guide RNA (gRNA), we employed two separate approaches using distinct gRNAs (Supplemental Fig. S1, *A* and *B*). Using the tool CCTop (17), we selected two gRNAs with optimal on-target efficiency and minimal off-target activity. hiPSCs were electroporated with plasmids encoding one of the selected gRNAs, alongside a Cas9/GFP expression vector. Subsequently, GFP-positive cells were isolated *via* FACS, enabling the selection and expansion of individual clones. To confirm successful gene editing, we performed Sanger sequencing on genomic regions flanking the gRNA target sites in both WT and putative KO hiPSCs. The obtained sequences were analyzed using the insertion-deletion (indel) mutation analysis software SYNTHEGO [Synthego Performance Analysis, ICE Analysis. 2019. v3.0. Synthego; [18.08.2023]]. Thereby compound heterozygous mutations were identified in two specific clones, resulting in frameshifts and premature stop codons on both alleles (Supplemental Fig. S1*C*). These clones were subsequently designated as UGCG KO1 and UGCG KO2.

**Fig. 1:**
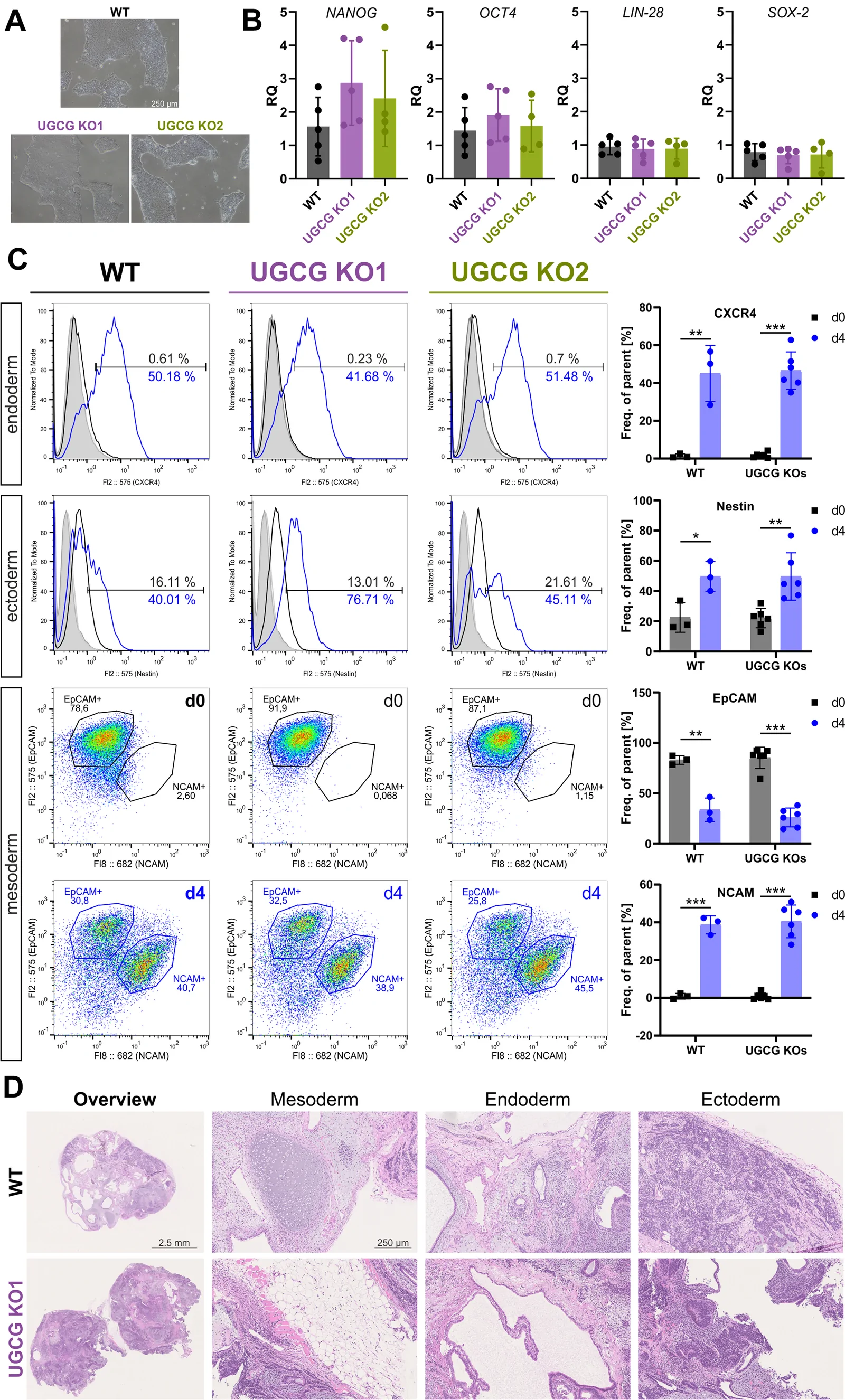
**Initial Characterization of glucosylceramide synthase-deficient (UGCG KO) hiPSCs.***A*, microscopic pictures presenting the morphology of wildtype (WT) and two different UGCG KO hiPSC lines. *B*, relative expression levels of characteristic stem cell markers assessed by qPCR. *C*, cell surface expressions of hiPSCs and derivatives differentiated into endo, ecto, or mesoderm. Flow cytometric analyses of WT and UGCG KO hiPSCs (d0) and their derivatives on day 4 of differentiation (d4) were assessed for each differentiation approach. One representative overlay histogram presents fluorescence intensities of viable cells on d0 (*black*) and d4 (*blue*). Gates were defined based on less than 1% positive cells in respective isotype controls (*gray*). Positive cells are shown as percentage of viable cell population. Bar graphs present frequency of parent in dependence of genotype on d0 and d4 of differentiation (n = 3–6). An unpaired Student's *t* test was performed for each target between d0 and d4 of each germlayer differentiation (∗*p* < 0.05; ∗∗*p* < 0.01, ∗∗∗*p* < 0.001). *D*, hematoxylin and eosin (HE) staining of teratoma originating from WT or UGCG KO1 hiPSCs. Besides an overview scan, meso, endo, and ectoderm specific structures are depicted at larger scale for both genotypes. hiPSC, human induced pluripotent stem cell; qPCR, quantitative polymerase chain reaction.

Both UGCG KO hiPSC lines maintained characteristic stem cell morphology (Fig. 1*A*) and proliferation rates compared to WT hiPSCs. In addition, mRNA expression levels of established pluripotency markers, including *NANOG*, *OCT4*, *LIN-28*, and *SOX-2*, were comparable between both UGCG KOs and WT hiPSCs (Fig. 1*B*). To further assess pluripotency, we evaluated the differentiation potential of the UGCG KO hiPSC lines into early progenitors of all three embryonic germ layers. We induced *in vitro* differentiation for 4 days towards endo-, ecto-, and mesodermal lineages. Flow cytometric analysis of lineage-specific markers in undifferentiated cells (d0) and their differentiated derivatives (d4) demonstrated comparable differentiation efficiencies between WT and UGCG KO cell lines (Fig. 1*C*). In detail, upon endodermal differentiation, the mean proportion of cells positive for the endodermal marker CXCR4 were 46.6% and 45% for UGCG KO and WT cells, respectively. In addition, the proportion of Nestin-positive cells significantly increased from mean values of 22.1% and 22.5% for UGCG KO and WT hiPSCs to degrees of 49.6% and 49.6% for UGCG KO and WT cells upon ectodermal differentiation, respectively. The analyzed hiPSC lines were almost completely EpCAM positive and NCAM-negative at d0 and mesodermal differentiation for 4 days caused a shift into two distinct populations, one positive for NCAM and negative for EpCAM and vice versa matching our previous observations (23), with rather similar efficacies for WT and KO cells lines. Finally, we assessed teratoma formation, a gold standard for validating pluripotency (42), by injecting WT and one of the two KO hiPSC lines (to minimize animal use) into immunocompromised mice. Teratoma derived from WT and UGCG KO1 hiPSCs were rather similar with regard to size, shape, and weight upon explantation after 7 to 9 weeks (data not shown). H&E staining of teratoma sections revealed distinct structures representative of all three embryonic germ layers both in WT and UGCG KO1 (Fig. 1*D*). Collectively, these analyses demonstrate that key hallmarks of pluripotency are preserved despite the genetic deletion of *UGCG* in hiPSCs.

### Genetic Manipulation of UGCG in hiPSCs Completely Abrogates Synthesis of GlcCer-Based GSLs

**Fig. 2:**
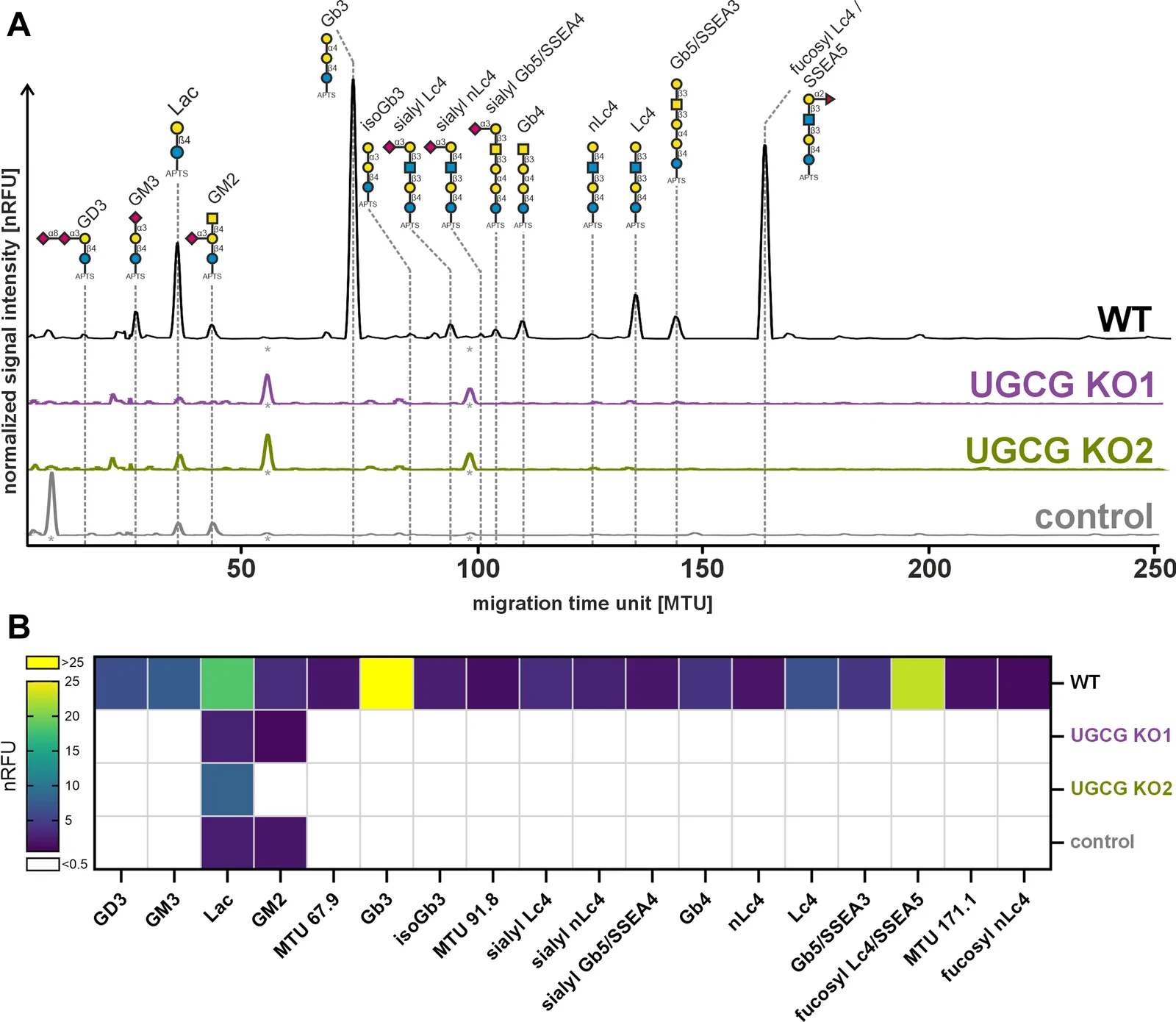
**GSL-derived glycan profiling of WT and UGCG KO hiPSCs glycosphingolipids (GSLs) were extracted from WT and UGCG KO cell pellets.** Subsequently, GSL-derived glycan profiles were assessed by multiplexed capillary gel electrophoresis coupled to laser-induced fluorescence detection (xCGE-LIF). *A*, representative electropherograms depicting normalized signal intensities of fluorescently labeled glycans in dependence of their migration time unit (MTU) for WT, UGCG KO1 and KO2 hiPSCs, and a negative-control which lacked cells, but was otherwise processed identically to the cell-containing samples. ∗ indicates signals originating from oligosaccharide impurities and are excluded from further analyses. Assigned GSL-derived glycans are depicted as pictograms with *purple diamond*: sialic acid; *yellow circle*: galactose; *blue square*: *N*-acetylglucosamine; *yellow square*: *N*-acetylgalactosamine; *red triangle*: fucose; *blue circle*: glucose. Symbolic representation of pictograms follows the guidelines of Symbol Nomenclature for Glycans (SNFG) (80). *B*, heatmap presents mean of normalized signal intensities (nRFU) of GSL-derived glycans exceeding a threshold of 2.5 nRFU (n = 3). GSL, glycosphingolipid; hiPSC, human induced pluripotent stem cell; UGCG, UDP-glucose ceramide glucosyltransferase gene.

The *UGCG* gene encodes a pivotal enzyme catalyzing the initial synthesis step of all GlcCer-based GSLs. To validate the functional consequences of our CRISPR/Cas9-mediated UGCG KO, we conducted a comprehensive GSL-derived glycan profiling in the engineered UGCG KO hiPSC lines, juxtaposing them with their WT counterparts. Therefore, we applied our well-established workflow for glycomic profiling using multiplexed capillary gel electrophoresis coupled to laser-induced fluorescence detection (xCGE-LIF) (24, 43, 44, 45, 46) (Fig. 2). Analysis of WT hiPSCs revealed a characteristic GSL-derived glycan profile, expressing globo- (SSEA3 and SSEA4) and lactoseries (Lc4 and SSEA5) GSLs, which are common markers of hiPSCs (46, 47, 48). In addition, the GSL-derived glycans Gb3 and lactose were detected, which are also commonly observed in mammalian cells. The aforementioned signals were not detected upon analysis of both UGCG KO hiPSC lines. Besides this, we observed weak signals corresponding to common GSL-derived glycans such as lactose; however, their levels were comparable to those in the cell-free negative control. The negative control, which was processed identically but without cells, exhibited GSL-derived glycan signals likely originating from bovine serum albumin, a known lipid carrier present in high concentrations in the commercial ceramide glycanase preparation and reaction buffer (Ceramide Glycanase Kit, Ludger) (49). Furthermore, all analyzed glycan profiles displayed signals corresponding to oligosaccharide impurities (labeled by asterisks, (50)) and were excluded from data evaluation. Taken together, glycomic profling revealed a complete loss of GlcCer-based GSLs in UGCG KO hiPSCs, confirming the functional deletion of the UGCG enzyme. Notably, *N*-glycan profiles were not altered due to the mutation of *UGCG* as they were comparable to the WT *N*-glycome (Supplemental Fig. S2).

### Kinase Signaling Pathways are Perturbed by GSL Deficiency

**Fig. 3:**
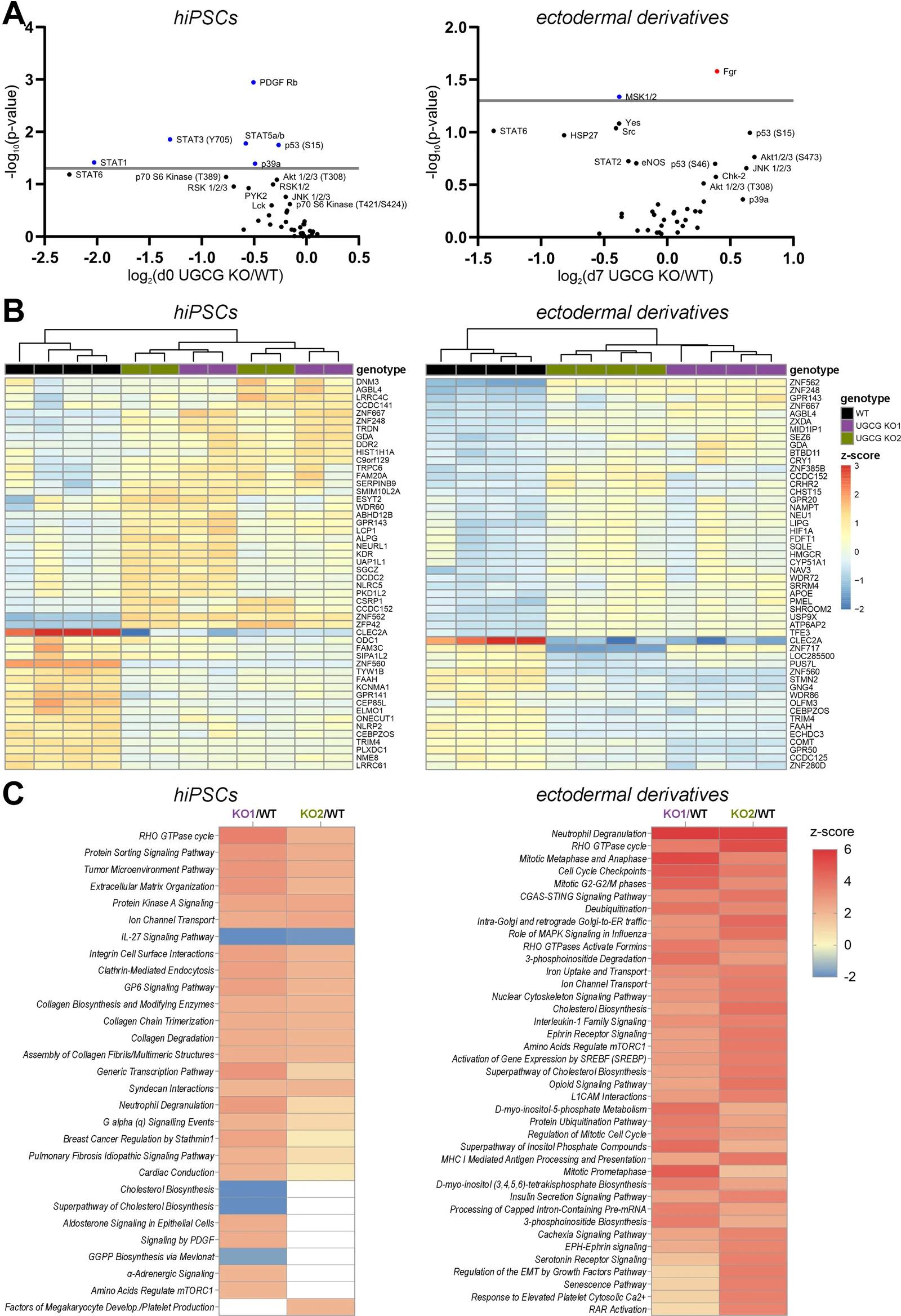
**Phospho-profiling and RNA Sequencing of UGCG KO and WT hiPSCs and their ectodermal derivatives.***A*, antibody array detection of phospho-kinase and targets of WT and UGCG KO hiPSCs and d7 ectodermal derivatives. *B*, heatmap displaying Z-scores representing gene expression levels in each sample relative to the mean expression across all samples. The heatmap includes the top 50 genes significantly deregulated in both UGCG KO lines compared to WT hiPSCs and day seven derivatives. Color scale indicates relative expression changes, with clustering highlighting expression patterns across conditions. *C*, heatmap of ingenuity pathway analysis (IPA) comparing enrichment of canonical pathways across experimental groups. Rows represent canonical pathways curated by IPA, and columns correspond to different conditions. Pathways are clustered to highlight similarities in activation or inhibition patterns. Predicted activation or inhibition of pathways, derived from IPA Z-scores, are shown by a divergent color scale: *red* signifies activation, *blue* signifies inhibition, and *white* indicates conditions where no Z-score could be calculated. hiPSC, human induced pluripotent stem cell; UGCG, UDP-glucose ceramide glucosyltransferase gene.

GSLs are well known to interact with receptors at the plasma membrane and modulate their signaling activity (5, 6). Especially during ectodermal differentiation a core structure switch from mainly globo and lacto-series to ganglio-series GSLs is described (51). Therefore, we analyzed the impact of GSL deficiency on signaling not only in hiPSCs, but also in day seven ectodermal derivatives. Using the Human Phospho-Kinase Array Kit (R&D Systems), we comparatively analyzed phosphorylation profiles of a set of different kinases and their protein substrates in WT and the herein described UGCG KO cells at the level of hiPSCs as well as upon 7 days of ectodermal differentiation (Fig. 3*A*). Differentiation was monitored by relative mRNA expression levels of pluripotency and lineage-specific markers (Supplemental Fig. S3). Three transcription factors belonging to the Signal Transducer and Activator of Transcription family (STAT1, STAT3, STAT5a/b), platelet derived growth factor receptor beta, tumor protein p53 (p53), and mitogen-activated protein kinase 14 (p38α) were significantly less phosphorylated in UGCG KOs compared to WT hiPSCs (Fig. 3*A* left). In hiPSC-derived ectodermal cells, Fgr phosphorylation was slightly increased in UGCG KO compared to WT, while MSK1/2 phosphorylation was decreased (Fig. 3*A* right). These findings imply a modulating role of GSLs on signaling pathways involved in stem cell maintenance and differentiation.

### UGCG Deficiency Leads to Widespread Changes in Gene Expression

To further complement our multi-omics analysis, we performed quantitative transcriptome profiling of WT and UGCG KO hiPSCs and their d7 derivatives using RNAseq (Fig. 3*B*). For each sample, sequenced reads were processed using DESeq2. 346 and 465 genes were identified to be significantly differentially expressed between both UGCG KOs compared to WT hiPSC lines at d0 and d7, respectively. The top 50 significantly regulted genes were ranked by Z-score (Fig. 3*B*). In the initial analysis of the top 50 differentially expressed genes, we focused on candidates consistently affected at both time points - day 0 hiPSCs and day seven ectodermal derivatives. This analysis revealed a marked reduction in the expression of C-type lectin domain family two member A in both UGCG KO clones at both stages. Also, several zinc-finger protein-encoding genes were among the most deregulated genes identified. Specifically, *ZNF248* and *ZNF562* were upregulated, while *ZNF560* was downregulated in both KO clones at both time points. Functional profiling using IPA (Qiagen) of all significantly regulated genes in both analyzed UGCG KO lines compared to WT, revealed enrichment of genes involved in canonical pathways comprising “Rho GTPase cycle”, “protein sorting signaling pathway”, “extracellular matrix organization”, and amongst others “IL-27 signaling” on hiPSC stage (Fig. 3*C* left). Similar enrichment analyses for d7 differentiated cells revealed enrichment of genes involved in canonical pathways comprising “Rho GTPase cycle”, “IL-1 family signaling”, and “activation of gene expression by SREBF” (Fig. 3*C* right).

### Lipidomic Profiling Reveals Increased SM Levels in UGCG-Deficient hiPSCs

**Fig. 4:**
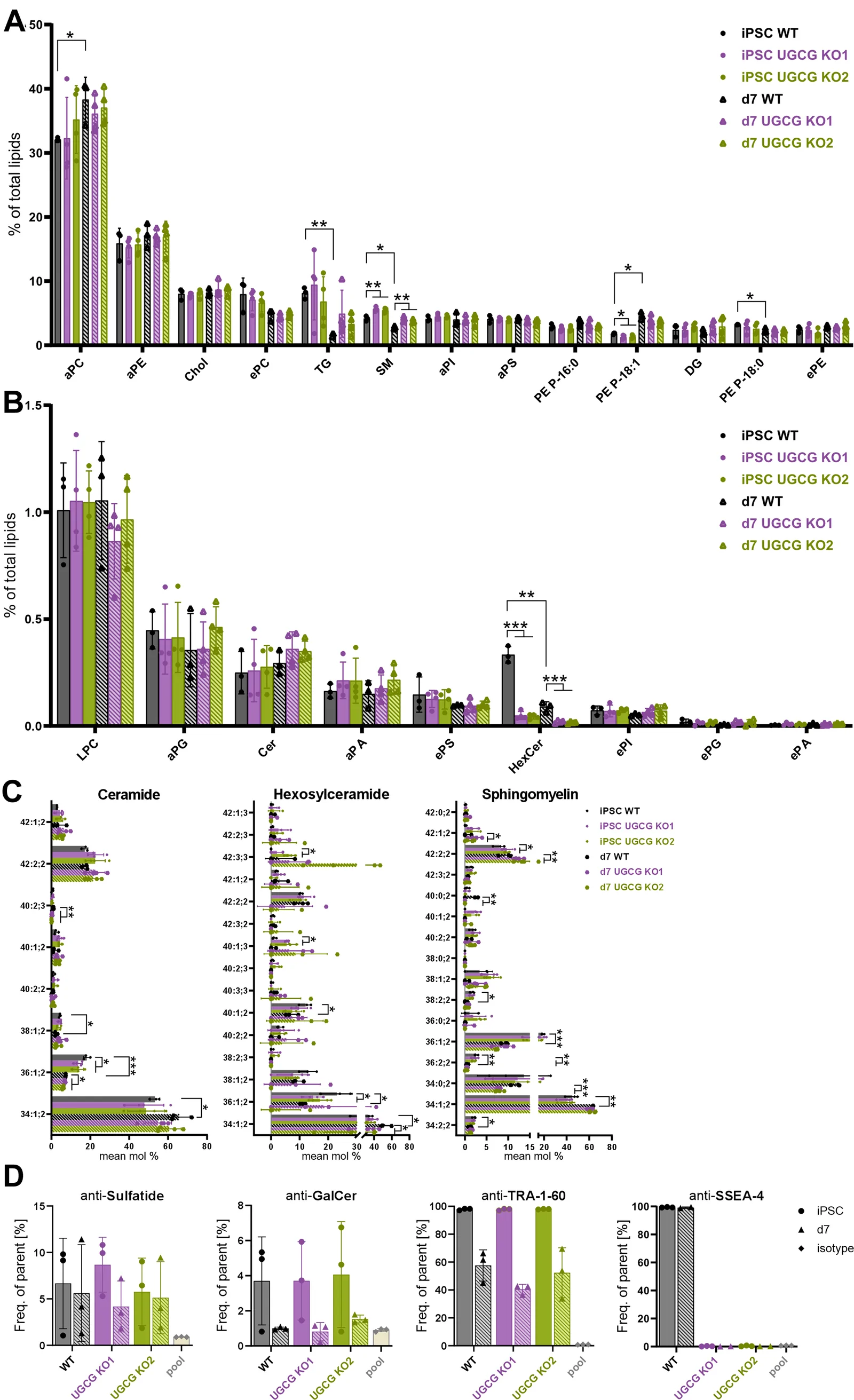
**Lipidomic profiling of UGCG KO and WT hiPSCs and their ectodermal derivatives.***A*, overview of all main classes of analyzed lipids exceeding 1% average abundance of total lipids. *B*, overview of all lipid classes below 1% average abundance of total lipids. The prefix *a* refers to di-acyl species, while the suffix *e* denotes glycerophospholipids containing either one even- and one odd-numbered fatty acyl chain or an ether lipid species. *C*, in-depth lipid subspecies analysis of ceramide, hexosylceramide, and sphingomyelin. Depicted are molecular percentages per lipid subspecies. Hatched areas present ectodermal derivatives of respective genotype. An unpaired Student's *t* test was performed between WT hiPSC and d7 derivatives, and between WT and UGCG KOs for each condition (∗*p* < 0.05; ∗∗*p* < 0.01, ∗∗∗*p* < 0.001). Lipid species annotations: x:y;z, where x = total number of C atoms, y = total number of double bonds, z = total number of hydroxylation. The numbers represent the sum of contributions from the sphingosine backbone and the N-acylated fatty acid. *D*, flow-cytometric analysis of WT and UGCG KO cells at iPSC state and after 7 days of ectodermal differentiation (d7). Gates were defined using pooled isotype controls (<1% positive cells, *gray*). Positive cells are expressed as a percentage of the viable cell population. Bar graphs show genotype-specific frequencies at d0 and d7 for each differentiation condition (n = 2–3). CE, cholesteryl esters; Cer, ceramide; Chol, cholesterol; DG, diacylglycerol; Hex2Cer, dihexosylceramide; HexCer, hexosylceramide; hiPSC, human induced pluripotent stem cell; LPC, lyso-phosphatidylcholin; PA, phosphatic acid; PC: phosphatidylcholine; PE, posphatidylethanolamine; PG, phosphatidylglycerol; PI, phosphatidylinositol; PS, phosphatidylserine; SM, sphingomyelin; TG, triacylglycerol; UGCG, UDP-glucose ceramide glucosyltransferase gene.

Lack of GlcCer-based GSLs is likely to interfere with lipid homeostasis. To assess the impact of UGCG deletion on the plasma membrane lipidome, we performed global lipidomic analysis by nano-electrospray ionization tandem MS (52). Overall, 24 distinct lipid classes were quantified relative to each other, with acyl-phosphatidyl-cholines (aPCs) emerging as the predominant lipid species in both hiPSCs and their d7 ectodermal derivatives (Fig. 4*A*). Notably, relative aPC levels were significantly higher at d7 in WT cells (≈38%) compared to their parental hiPSCs (≈32%). However, the UGCG KO did not result in any significant changes in aPC levels (Fig. 4*A*). Further abundant lipid species, inlcuding acyl-phosphatidyl-ethanolamines (≈15–17%) and cholesterol (Chol, ≈8%), exhibited consistent total levels irrespective of the genotype or ectodermal differentiation. Relative levels of total ether-phosphatidylcholines displayed a noticeable but nonsignificant trend toward reduced levels in d7 ectodermal cells, with no discernible differences observed between WT and UGCG KO cells. We also noted that relative levels of phosphatidylethanolamines PE P-18:1 were significantly increased from 1.8% to 4.6% in WT upon ectodermal differentiation (Fig. 4*A*). Relative levels of total TAG, of PE P-18:0, and of SM were significantly lower in d7 WT cells (≈6% reduction of TG, ≈1% reduction of PE P-18:0, and ≈1.5% reduction of SM) compared to hiPSCs. Remarkably, total SM levels were significantly elevated in both KO cell lines compared to WT, regardless of analysis at d0 or d7. Since SMs are directly synthesized from ceramides, deletion of UGCG - which blocks glycan attachment to ceramide - would be expected to also cause ceramide accumulation in the KO cell lines. However, ceramide levels were comparable between WT and UGCG KOs and presented only ≈0.25% of total lipids (Fig. 4*B*). The MS approach included only two glycosylated lipid species: HexCer and Hex2Cer. These GSLs comprised less than 1% of total lipids in WT samples but were almost absent in both UGCG KOs. Specifically Hex2Cer levels were below detection limits in UGCG KOs and therefore not shown. In WT cells, levels of HexCer and Hex2Cer were significantly reduced following seven days of ectodermal differentiation (Fig. 4*B*), potentially implying the generation of more complex type glycans. To further assess whether GalCer or related species were compensatorily increased upon UGCG deletion, we performed flow cytometric analysis of GalCer and sulfatide, using TRA-1-60 and SSEA-4 as internal pluripotency controls (Fig. 4*D*). TRA-1-60 surface staining decreased from nearly 100% positive cells in the iPSC state to approximately 50% after 7 days of ectodermal differentiation in all three cell lines, with no genotype-specific differences. By contrast, SSEA-4 was detectable only in WT cells, together with the xCGE-LIF data further confirming the functional impairment of UGCG in the KO lines. Consistent with the lipidomic data, GalCer and sulfatide were detected only at low surface levels and showed no genotype-specific differences.

Furthermore, we conducted quantitative analyses of lipid subclasses, specifically examining variations in fatty acid chain length and degree of saturation across all identified lipid species. Subclass analysis of ceramide and SM revealed an increase in species with shorter fatty acid chain lengths (34:1,2 or 36:1,2) in WT cells, while species with longer fatty acids (40:1,2 or 40:2,2 or 42:1,2 or 42:2,2) were more abundant in the mutant cell lines (Fig. 4*C*). Notably, the increase in mutants compared to WT was particularly pronounced for more saturated SMs (*e*.*g*. 40:1,2 exhibited a stronger increase in mutants than 40:2,2; or 42:1,2 higher enrichment in mutants than 42:2,2) (Fig. 4*C*).Together, the lipidomic profiling revealed overall changes in lipid content resulting from ectodermal differentiation of WT hiPSCs, as well as genotype-induced alterations originating from the UGCG KO.

### Depletion of UGCG Is Associated with Proteomic Alterations in hiPSC-Derived Teratoma

**Fig. 5:**
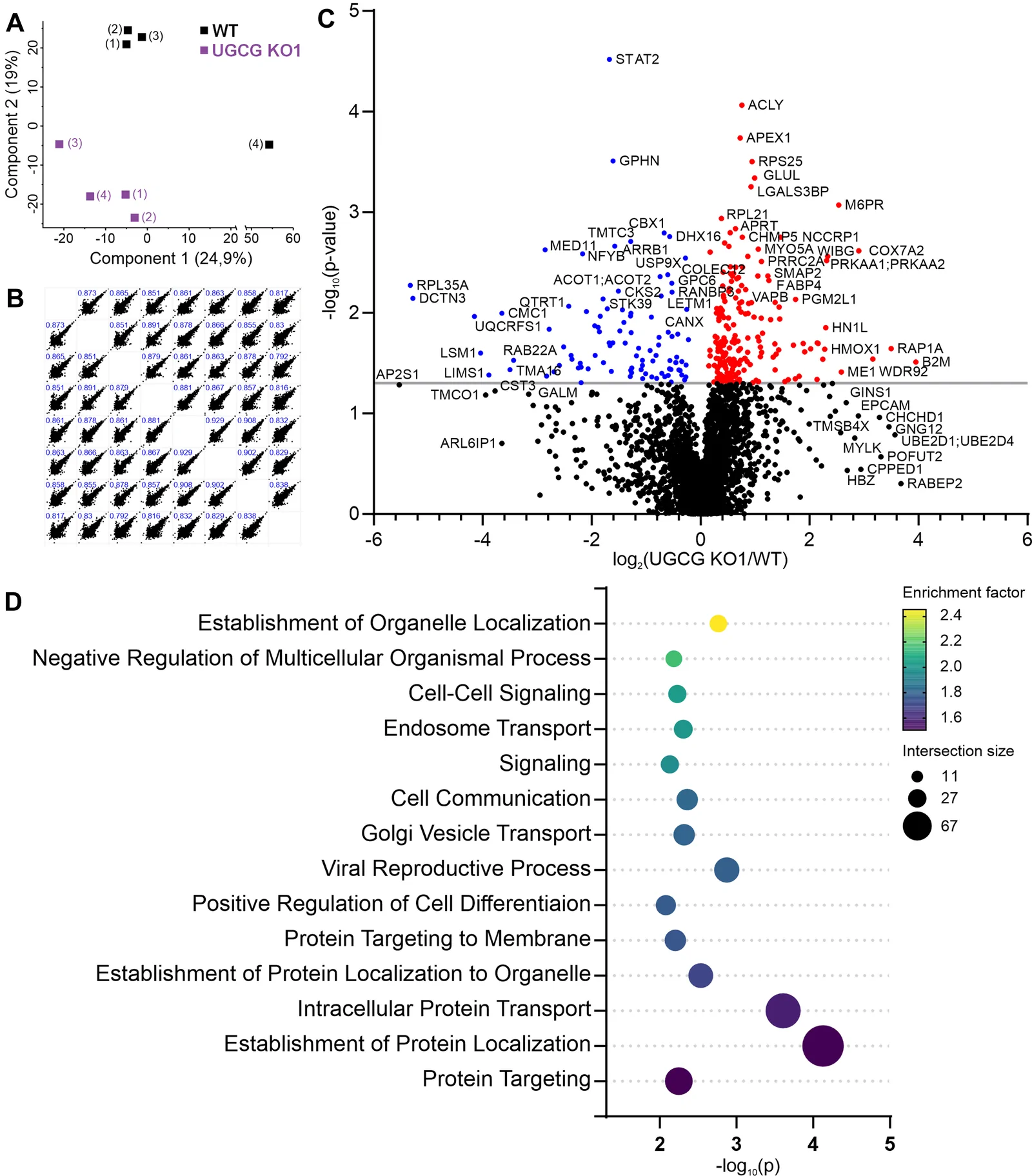
**Whole proteomic profiling of teratoma *via* mass spectrometry.***A*, PCA analysis of generated whole proteomic data. *B*, Pearson correlation of respective data sets. *C*, volcano plot displays ratio of protein levels (fold change) detected in UGCG KO1- *versus* WT-derived teratoma. Significantly regulated proteins are highlighted in *blue* (down regulation in UGCG KO) and *red* (up regulation in UGCG KO). *D*, Fisher enrichment analysis of biological pathways according to the GOBP database. GOBP, Gene Ontology Biological Process; PCA, principal component analysis; UGCG, UDP-glucose ceramide glucosyltransferase gene.

Teratomas serve as an ideal *in vivo* model to recapitulate early developmental differentiation of human pluripotent stem cells (53). To investigate whether the absence of GSLs interferes with pluripotent stem cell differentiation, we performed proteomic profiling of teratoma derived from WT iPSCs and one UGCG KO clone. Quantitative comparison of the proteomes of four different WT- and four different UGCG KO-derived teratoma by LC-MS/MS led to the identification of 6107 distinct proteins in total with 3152 proteins left upon filtering out reverse database hits, proteins only identified by site, potential contaminants, and restriction to proteins identified in at least three out of four replicates in at least one group. A principal component analysis resulted in distinct clustering of WT and UGCG KO hiPSC-derived teratoma samples, demonstrating a clear global effect of *UGCG* deletion on the teratoma proteome (Fig. 5*A*). Pairwise comparisons resulted in Pearson correlations above 0.8 for all samples implying a very high correlation between WT and UGCG KO derived teratoma data sets (Fig. 5*B*). Of the 3152 proteins selected for further analysis, 205 were significantly upregulated and 98 were downregulated in UGCG KO-derived teratoma proteomes compared to WT-derived teratoma proteomes (Fig. 5*C*). We observed significant enrichment of differentially expressed proteins across a range of biological pathways (Fig. 5*D*). Closer examination revealed that the UGCG KO affects a network of interconnected cellular processes. Notably, many of the top enriched pathways, including “establishment of organelle localization”, “endosome transport”, “protein targeting to membrane”, “establishment of protein localization to organelle”, “intracellular protein transport”, and “protein targeting” are related to intracellular trafficking and protein localization (Fig. 5*D*). In addition, pathways involved in cell communication and signaling, such as “cell-cell signaling”, “signaling”, and “cell communication” were also significantly enriched. Representative gene sets for selected pathways are visualized in individual volcano plots (Supplemental Fig. S9). Notably, we observed elevated protein levels of ATP-citrate lyase, an enzyme that generates acetyl-CoA for fatty acid and cholesterol biosynthesis in UGCG KO conditions compared to WT controls. Conversely, protein levels of acyl-CoA thioesterase 1 (ACOT1)—an enzyme that catalyzes the hydrolysis of acyl-CoA to CoA and free fatty acids—were significantly reduced in UGCG KO conditions. Both regulatory changes are consistent with a metabolic shift towards increased membrane lipid synthesis in UGCG KO cells. Supporting this notion, Fisher's exact enrichment analysis revealed a significant enrichment of proteins involved in the positive regulation of lipid biosynthetic processes (Supplemental Fig. S8). Furthermore, consistent with the reduced phosphorylation of STAT family proteins observed in UGCG KO cells, we also detected decreased total protein levels of STAT2 in these cells. Taken together, comprehensive proteomic profiling of UGCG KO1 and WT hiPSC-derived teratomas revealed significant alterations in the abundance of 303 proteins, encompassing those involved in lipid metabolism, signaling pathways, and other cellular processes, thus providing first insights into the broader impact of UGCG deficiency on cellular differentiation and function.

## Discussion

GSLs are important cell surface glycoconjugates with diverse functions in the mammalian system (1). A previous study has shown in a mouse model that complete loss of GlcCer-based GSL synthesis due to *Ugcg* gene disruption leads to embryonic lethality during gastrulation (10). Encouraged by these findings, we focused our investigation on hiPSCs, to further elucidate the role of GSLs in human pluripotency and early differentiation stages. Therefore, we generated two UGCG KO hiPSC lines from the parental cell line using CRISPR/Cas9 with two distinct gRNAs. We employed two separate approaches to distinguish between UGCG-specific effects and potential off-target effects, thereby ensuring robustness of our findings. Both UGCG KO lines showed complete lack of GlcCer-based GSLs, proving functional enzyme inactivation by the genetic manipulation. Nevertheless, common pluripotency marker (*NANOG*, *OCT4*, *LIN28*, *SOX-2*) expression and the capacity for directed *in vitro* differentiation into early meso-, endo-, and ectodermal progenitors remained unaffected by the UGCG KO. We furthermore generated teratoma derived from UGCG KO and WT hiPSCs by subcutaneous injection into immunocompromised mice. Teratoma formation is considered the gold standard for assessing the differentiation potential of pluripotent stem cells in a complex, multicellular environment (53). UGCG-deficient hiPSCs successfully differentiated into derivatives of all three germ layers without apparent differences compared to their WT counterparts. Consistent with our findings, Ugcg-deficient mouse embryos successfully underwent early embryogenesis, including differentiation into primitive germ layers and murine embryonic stem cells lacking Ugcg also retained their ability to differentiate into all three embryonic germ layers *in vitro* (10).

These findings suggest that GSLs may be dispensable for maintaining pluripotency in human pluripotent stem cells (hPSCs). However, it also raises the intriguing question of why hPSCs specifically and abundantly express GSLs belonging to the globoseries [SSEA-3, -4 (47) and Globo H (54)] and lactoseries [Lc4 (46), SSEA-5 (48), fucosyl-Lc4 (55) and sialyl-Lc4 (56)]. All aforementioned GSLs share a motif, synthesized by the β1,3-galactosyltransferase B3GALT5. This enzyme is specifically expressed in hPSCs and downregulated during differentiation (46, 57). Corroborating our observations, recent studies demonstrated that B3GALT5 KO, resulting in complete lack of hPSC-specific GSL species, did not impair their pluripotency, neither affecting marker gene expression nor *in vitro* or *in vivo* differentiation capacity (58). However, B3GALT5-deficient hPSCs shifted their GSL profile towards neolactoseries GSLs like SSEA-1 (58), typically associated with murine PSCs (59). Murine PSC are known to exhibit a more naïve pluripotent state compared to hPSCs (60), and deletion of B3GALT5 in hPSCs was suggested to facilitate the transition from the primed to the naïve state (58).

Motivated by these findings that changing the GSL profile might have specific molecular effects on cultured hPSCs, we characterized our GSL-deficient hiPSC models by different omics approaches. GSLs are highly important in the nervous system and sialylated GSLs (gangliosides) are the major glycoconjugates of neurons (61). Accordingly, genetic interference with ganglioside synthesis in mice is often associated with diverse neurological defects (62). Therefore, we also included UGCG KO hiPSC-derived d7 ectodermal derivatives in our study.

GSLs constitute a major fraction of membrane lipids and their absence likely disrupts lipid homeostasis in hiPSCs and their derivatives. To investigate the impact of UGCG depletion on cellular lipids, we performed comprehensive lipidomic profiling. Because UGCG catalyzes the transfer of glucose to ceramide (63), UGCG deficiency might be expected to alter ceramide-derived GSLs such as GalCer or sulfatide, or to increase ceramide levels. However, we noted that ceramide levels were unchanged in UGCG KO hiPSCs and d7 ectodermal derivatives.

Similarly, surface staining showed comparable levels of GalCer and sulfatide in UGCG KO and WT cells. Although these findings raise the possibility that GalCer-based GSLs may partially compensate for the loss of GlcCer-derived GSLs, this was not further investigated in the present study. In addition, because GSL glycan structures share similarities with O-glycans, a compensatory contribution of altered O-glycosylation cannot be excluded. Notably, SM, a direct ceramide derivative, was significantly increased in both cell lines, suggesting a rerouting of ceramide toward SM upon UGCG depletion. The increased SM levels suggest that, in the absence of UGCG, available ceramide is redirected to SM synthesis *via* SM synthases (64, 65). This reaction transfers a phosphorylcholine group from PC to ceramide, producing SM and DAG as a byproduct. SMs are critical for maintaining membrane integrity and regulating cellular signaling (66). In addition, GlcCer also serves as a donor substrate for glucosylated cholesterol, a recently described steryl glycoside in mammalian tissues and plasma (67), but the potential effect of UGCG depletion was not further evaluated in this study.

GSLs are well known to directly interact with and thereby modulate the function of receptor tyrosine kinase-mediated signaling pathways (62), as demonstrated for the EGFR (6) and the insulin receptor (68). To assess a possible effect on kinase-dependent signaling pathways in UGCG KO hiPSCs and derived ectodermal derivatives, we profiled the phosphorylation state of several kinases and their downstream targets. Phosphorylation of numerous different STAT-family proteins (STAT1, STAT3, and STAT5a/b) was significantly reduced in GSL-deficient hiPSCs. STATs are targets of various membrane localized Janus kinases and STAT3 was shown to be activated by growth factor receptors like EGFR (69). Based on these findings it can be hypothesized that complete lack of GlcCer-based GSLs negatively interfered with kinase activity. Previously it was shown that STAT3 plays a critical role in facilitating reprogramming of human somatic cells, as reduced reprogramming efficiencies of primary skin fibroblasts of patients with dominant negative mutations in *STAT3* were observed (70). Furthermore, it was described that Janus kinase-STAT signaling is involved in the control of stem cells during hematopoiesis in the bone marrow and neurogenesis in the developing cortex (71, 72).

To determine whether the observed changes in membrane lipid composition and protein phosphorylation within signaling pathways are reflected at the gene expression level, we conducted comparative transcriptomic profiling of WT and UGCG KO cells at d0 and d7. Functional pathway analysis indicates that UGCG loss affects key signaling pathways in hiPSCs, including “protein sorting signaling pathway”, “protein kinase A signaling”, “IL-27 signaling pathway”, and “GP6 signaling pathway” (5, 73, 74, 75).

To assess whether the observed effects are exclusive to the *in vitro* culture system or can also be recapitulated in a more physiologically relevant context, we performed quantitative proteomic analysis of teratomas derived from UGCG KO and WT hiPSCs. These teratoma were generated by subcutaneous injection into immunocompromised mice and harvested after 7 to 9 weeks of *in vivo* growth. Comparative proteomic profiling revealed distinct alterations in the teratoma proteomes upon GSL depletion. Pathway enrichment analysis highlighted changes in intercellular communication and signaling cascades, consistent with findings from our phospho-kinase array and transcriptomic datasets.

GSL deficiency also affected pathways involved in intracellular transport, particularly vesicular trafficking processes associated with the Golgi apparatus and endosomes. These findings suggest broader disruptions in membrane organization and the spatial distribution of proteins and organelles, consistent with the established role of GSLs in membrane architecture and vesicular trafficking (76, 77, 78). This is further supported by our transcriptomic data, which revealed enrichment of gene sets related to “protein sorting signaling pathways”, “extracellular matrix organization”, and “clathrin-mediated endocytosis”. These molecular alterations may, at least in part, be linked to the altered lipid composition in UGCG-deficient hiPSCs, specifically the observed increase in SM levels.

## Conclusion

To our knowledge, this study presents the first human UGCG KO iPSC model and applies a multi-omics approach to investigate the functional role of GSLs in early developmental processes *in vitro*. We show that GlcCer-based GSL deficiency does not impair core pluripotency or directed differentiation capacity. Instead, it induces distinct molecular alterations across multiple cellular systems. These results reveal that GlcCer-based GSLs contribute to cellular adaptation and robustness during early developmental processes rather than act as indispensable determinants. Collectively, our study provides new insight into the regulatory roles of GSLs in a human stem cell model.

## Data Availability

All data are available in the main text or the supplemental files. The mass spectrometry proteomics data have been deposited to the ProteomeXchange Consortium *via* the PRIDE (79) partner repository with the dataset identifier PXD071827. The mass spectrometry lipidomics data are available upon request. The RNA Sequencing data have been deposited to the European Nucleotide Archive (ENA) with the accession number PRJEB105215.

## Supplemental Data

This article contains supplemental data.

## Conflict of Interest

The authors declare that they have no conflicts of interest with the contents of this article.
