# Supplementary material for: Integrated Multi-Omics Analysis Uncovers Molecular Dysregulation in Glucosylceramide Synthase-Deficient Human Induced Pluripotent Stem Cells
Source: Mol Cell Proteomics. 2026 Jul 23;25(8):101630. doi: 10.1016/j.mcpro.2026.101630 (PMC13520899; doi:10.1016/j.mcpro.2026.101630)
Supplement: Supplementary file 1 [file mmc1.docx]

## Beimdiek-et-al_UGCG-Multiomics

# Supplementary Figures

**Supplementary Figure 1:** **Generation of glucosylceramide-synthase depleted (UGCG KO) induced pluripotent stem cell lines (iPSCs).**

(A) Workflow for gene editing of the UGCG locus in human induced pluripotent stem cells (iPSCs) Phoenix. (B) Co-electroporation of a sgRNA harbouring and a Cas9-GFP harbouring plasmid. (C) Clone screening was performed via Sanger Sequencing and subsequent indel analysis using the SYNTHEGO ICE analysis tool.

**Supplementary Figure 2: N-glycome of WT and UGCG KO hiPSCs**

Examination of whole cell N-glycosylation profiles by capillary gel electrophoresis coupled to laser-induced fluorescence detection (xCGE-LIF) revealed no significant differences between Phoenix WT and UGCG KO hiPSCs. Assigned N-glycans are depicted as pictograms with purple diamond: sialic acid; yellow circle: galactose; blue square: N-acetylglucosamine; green circle: mannose, red triangle: fucose; blue circle: glucose. Symbolic representation of pictograms follows the guidelines of Symbol Nomenclature for Glycans (SNFG).

**Supplementary Figure 3**: **Stem cell and differentiation markers during ectodermal differentiation**

Phoenix, UGCG KO1, and UGCG KO2 hiPSCs and their ectodermal derivatives were analyzed on d0 and d7 of differentiation. mRNA expression levels were determined by qPCR analysis. Depicted are 3 replicates for each biological group (n=3).

**Supplementary Figure 4:**

Gene raw counts of pluripotency, ectodermal, and EMT marker.

**Supplementary Figure 5:**

(A) Volcano plot displaying significantly regulated genes between d7 ectodermal derivatives compared to WT iPSC. (B) Volcano plot displaying significantly regulated genes between d7 ectodermal derivatives compared to UGCG KO1 iPSC. (C) Volcano plot displaying significantly regulated genes between d7 ectodermal derivatives compared to UGCG KO2 iPSC.

**Supplementary Figure 6:**

(A) Fisher Exact Enrichment analyses of differentially expressed genes between UGCG KO and WT iPSCs. Depicted are enrichment factors in grey and p-values in red.

**Supplementary Figure 7**:

(A) Fisher Exact Enrichment analyses of differentially expressed genes between UGCG KO and WT d7 ectodermal derivatives. Depicted are enrichment factors in grey and p-values in red.

**Supplementary Figure 8:**

Fisher Exact Enrichment analyses of differentially expressed proteins between UGCG KO1 and WT derived teratoma proteomes. Depicted are enrichment factors in grey and p-values in red.

**Supplementary Figure 9:**

Volcano plots of genes of indicated pathways in proteomic analysis of teratoma.

**Supplementary Figure 10:** Annotated fragmentation spectra of GPX5.

# Supplementary Tables

Supplementary Table S1: gRNA for CRISPR/Cas9 approach

| Name | gRNA Sequence (5’ 🡪 3’) | PAM | CRISPRrater - CCTop |
| --- | --- | --- | --- |
| I - 5673 | TTAGGATCTACCCCTTTCAG | TGG | 0.847 |
| II - 5987 | TCCTAACTTAATCAACAACC | TGG | 0.43 |

Supplementary Table S2: Clone Screening

| Name | Target | Allele 1 | Allele 2 | Conclusion |
| --- | --- | --- | --- | --- |
| I – UGCG KO1 | Exon 2 | 2 bp del | 10 bp del | Bi-allelic frameshift |
| II – UGCG KO2 | Exon 2 | 7 bp del | 8 bp del | Bi-allelic frameshift |

Supplementary Table S3: Primer (CRISPR Screen, qPCR)

| Application | Target | Sequence (5’ 🡪 3’) | Amplicon size [bp] | Efficiency [%] | NGS |
| --- | --- | --- | --- | --- | --- |
| *CRISPR/Cas9 clone screening* | UGCG exon 2 – gDNA_fw | GATAGCTTGTCAGTGCTTTG | 496 | - | ✓ |
|  | UGCG exon 2 – gDNA_rev | TTCAAACTACCAGGCACTTG |  | - | ✓ |
|  | UGCG exon 2 – cDNA_fw | TATCATCTACACCCGATTACACCTC | 167 | - | ✓ |
|  | UGCG exon 2 – cDNA_rev | GGAGCACTTCATATTTGGGATAATC |  | - | ✓ |
| *qPCR analysis* | PPIA_fw | CACCGTGTTCTTCGACATTGC | 191 | 95 | ✓ |
|  | PPIA_rev | TGAAGTCACCACCCTGACACA |  |  |  |
|  | RPLP0_fw | TGGTCATCCAGCAGGTGTTCGA | 119 | 103 | ✓ |
|  | RPLP0_rev | ACAGACACTGGCAACATTGCGG |  |  |  |
|  | Oct-4_fw | AGAAGGAGAAGTUGGAGCAA | 138 | 98 | ✓ |
|  | Oct-4_rev | CTTCCCAAATAGAACCCCCA |  |  |  |
|  | Nanog_fw | TACCTCAGCCTCCAGCAGAT | 146 | 94 | ✓ |
|  | Nanog_rev | TGCGTCACACCATTGCTATT |  |  |  |
|  | SOX-2_fw | ATGGAGAAAACCCGGTACGC | 112 | 108 | ✓ |
|  | SOX-2_rev | TTTTGCGTGAGTGTGGATGG |  |  |  |
|  | LIN28_fw | TTGAGGAGCAGGCAGAGTGG | 162 | 93 | ✓ |
|  | LIN28_rev | TGCATTTGGACAGAGCATGG |  |  |  |
|  | Pax6_fw | GCAACATCCGTGGAGAAAACTC | 249 | 112 | ✓ |
|  | Pax6_rev | GCTCGAATATGGGGCTCTGAA |  |  |  |
|  | MAP2_fw | CGAAGCGCCAATGGATTCC | 161 | 110 | ✓ |
|  | MAP2_rev | TGAACTATCCTTGCAGACACCT |  |  |  |
|  | EGFR_fw | GCACCTACGGATGCACTGGG | 198 | 91 | ✓ |
|  | EGFR_rev | CCACAAGCTCCCTCTCCTGC |  |  |  |

Supplementary Table S4: Antibodies used for flow cytometry

| Target | Host species (isotype) | Fluoro-chrome | Dilution | Manufacturer |
| --- | --- | --- | --- | --- |
| Primary antibodies |  |  |  |  |
| α-CXCR4 | recomb. human IgG1 | PE | 1:50 | Miltenyi Biotec (130-117-504) |
| α-EpCAM | recomb. human IgG1 | PE | 1:50 | Miltenyi Biotec (130-111-116) |
| α-GalCer | mouse IgG3 | FITC | 1:50 | Merck (FCMAB312F) |
| α-NCAM | mouse IgG_2b_ | APC | 1:100 | BD Biosciences (341027) |
| α-Nestin | Mouse IgG1 κ | PE | 1:20 | Biolegend (656805) |
| α-sulfatide | rat IgG2b κ | - | 1:100 | 2BScientific (AB02985-8-1) |
| α-SSEA-4 | recomb. human IgG1 | APC | 1:10 | Miltenyi Biotec (130-098-347) |
| α-TRA-1-60 | recomb. human IgG1 | PE | 1:50 | Miltenyi Biotec (130-122-965) |
| Secondary antibodies |  |  |  |  |
| α-mouse IgG | goat | AF488 | 1:100 | Molecular Probes (A11029) |
| α-mouse IgG2b | mouse | APC | 1:100 | BD Biosciences (565381) |
| α-rat IgG2 | goat | AF647 | 1:500 | Cell Signaling (NEB) |
| REA control APC | recomb. human | APC | 1:50 | Miltenyi Biotec (130-104-615) |
| REA control PE | recomb. human | PE | 1:50 | Miltenyi Biotec (130-104-613) |

Supplementary Table 5: Lipidomics MS settings and scan procedures

Supplementary_TableS5_Lipidomics_Settings_MS.xlsx

Supplementary Table 6: Teratoma whole proteomic data set

Supplementary_TableS6_Teratoma_Proteomics_identified_proteins.xlsx
